# A framework for measuring the cost to families of caring for children’s health: the design, methodology, and study population of the r-Kids study

**DOI:** 10.1186/s12887-023-03893-7

**Published:** 2023-03-20

**Authors:** Joanna E. Bulkley, Alexandra M. Varga, John F. Dickerson, Phil Crawford, Lisa A. Croen, Yihe G. Daida, Eric Fombonne, Brigit Hatch, April Lee, Maria Massolo, Katherine Vaughn, Frances L. Lynch

**Affiliations:** 1 Kaiser Permanente Center for Health Research, 3800 North Interstate Avenue, Portland, OR 97227-1110 USA; 2 Kaiser Permanente Division of Research, Oakland, CA USA; 3 Kaiser Permanente Center for Integrated Health Care Research, Honolulu, HI USA; 4 Oregon Health & Science University, Portland, OR USA; 5 OCHIN, Inc, Portland, OR USA

**Keywords:** health economics, employment, financial impact, autism, asthma, stress, ethnicity, child health

## Abstract

**Background:**

All families experience financial and time costs related to caring for their children’s health. Understanding the economic burden faced by families of children with chronic health conditions (CHC) is crucial for designing effective policies to support families.

**Methods:**

In this prospective study we used electronic health records to identify children between 3 and 17 years old with autism spectrum disorder (ASD), asthma, or neither (control) from three Kaiser Permanente regions and several community health centers in the OCHIN network. We oversampled children from racial and ethnic minority groups. Parent/guardian respondents completed surveys three times, approximately four months apart. The surveys included the Family Economic Impact Inventory (measuring financial, time, and employment costs of caring for a child’s health), and standardized measures of children’s quality of life, behavioral problems, and symptom severity for children with ASD or asthma. We also assessed parenting stress and parent physical and mental health. All materials were provided in English and Spanish.

**Results:**

Of the 1,461 families that enrolled (564 ASD, 468 asthma, 429 control), children were predominantly male (79%), with a mean age of 9.0 years, and racially and ethnically diverse (43% non-Hispanic white; 22% Hispanic; 35% Asian, Black, Native Hawaiian, or another race/ethnicity). The majority of survey respondents were female (86%), had a college degree (62%), and were married/partnered (79%). ASD group respondents were less likely to be employed (73%) than those in the asthma or control groups (both 80%; *p* = .023). Only 32% of the control group reported a household income ≤ $4,000/month compared with 41% of asthma and 38% of ASD families (*p* = .006).

**Conclusions:**

Utilizing a novel measure assessing family economic burden, we successfully collected survey responses from a large and diverse sample of families. Drawing upon the conceptual framework, survey measures, and self-report data described herein we will conduct future analyses to examine the economic burdens related to CHC and the incremental differences in these burdens between health groups. This information will help policy makers to design more equitable health and social policies that could reduce the burden on families.

**Supplementary Information:**

The online version contains supplementary material available at 10.1186/s12887-023-03893-7.

## Background

All families incur costs related to caring for their children’s health, whether through direct financial payments for medical care or through costs related to caring for their children when they are sick at home. In the United States approximately 38% of children have one or more current or lifelong chronic health conditions and these increase the economic burden to families [1]. To date, most research on the economic impact of child health conditions has focused on the direct healthcare costs to insurers and health systems [2–6]. The costs families experience in caring for a child’s health, and in particular caring for a child with a chronic health condition, are multifactorial and include direct financial costs, as well as other economic burdens such as time spent caring for their child’s health and related employment difficulties for parents. Economic burdens may also vary based on the child's specific health condition and across age groups (e.g., early childhood, school age, adolescent) as there is reason to hypothesize that specific health conditions may impact families differently as a child ages.

Although a number of studies have estimated one or two types of costs families experience, few have examined a broad range of family costs for any health condition. Several studies have used data from the National Survey of Children with Special Health Care Needs (NSCSHCN) [7–9] to estimate financial burden for families. While the NSCSHCN has significant strengths, questions about financial costs are broad (e.g. asking whether the family has out-of-pocket expenditures greater than $1000 in a year); and parents are asked to recall costs over a 12-month period, which can be challenging [10, 11]. In addition, the NSCSHCN data are solely parent self-report with no clinical validation of parent reported diagnoses. Finally, the NSCSHCN only includes families of children with special health care needs, so comparisons to typically developing children, or children with common physical health conditions (e.g., asthma) are not possible.

The r-Kids study was designed to address these gaps in the literature by collecting comprehensive and detailed data on the economic burden to families caring for a child’s health by examining costs from childhood through adolescence for families of children with autism spectrum disorder (ASD), asthma, and with neither health condition.

We focus on ASD for several reasons. The prevalence of autism spectrum disorder (ASD) is on the rise. Currently, the Centers for Disease Control estimates that about one in 44 children in the United States (2.3%) can be classified as having ASD [12]. In addition, several studies suggest that families of children with ASD face a range of financial costs [2–6, 13–16], such as out-of-pocket expenses for medical and other rehabilitative services, as well as time costs resulting from time demands related to managing the child’s ASD (e.g., coordinating school services or implementing behavioral management programs at home) [17, 18]. The caregiving demands associated with parenting a child with ASD can lead to employment difficulties for parents [2, 14, 15, 19–21]. In addition, these demands are often very stressful for families [22, 23], and the cumulative burden of the financial costs, time costs, and employment difficulties can lead to significant economic difficulties for families [17, 18].

Families of children with asthma were included in the study because we wanted to compare the costs that families of children with ASD experience to those experienced by families of children with other types of CHC. We chose to focus on asthma as a comparison condition because it is one of the most common CHCs in childhood and often requires significant medical care and other costs to families. In addition, our approach of studying a broad range of costs related to child health is potentially relevant to many pediatric health conditions such as diabetes, epilepsy, cystic fibrosis, or other ongoing health conditions.

The primary purpose of this report is to describe a conceptual approach to measuring costs to families caring for a child with a chronic health condition and to provide a description of the study design and methods, measures utilized, and planned outcome variables. In addition, we report the characteristics of the population and the enrolled study sample.

## Methods

The r-Kids study is an observational study assessing family financial, time, and employment costs, as well as the child’s quality of life and behavioral adjustment; parent physical and mental health; and household characteristics. We recruited parents/guardians of children with ASD, asthma, and neither condition (control) to complete surveys three times, approximately four months apart, resulting in the capture of information covering approximately one year. Survey data collection started in November 2017 and ended in January 2020, prior to the beginning of the COVID-19 pandemic.

### Participants and setting

All parents or guardians of children between 3 and 16.5 years of age from three non-profit integrated health care systems— (1) Kaiser Permanente Northwest (Oregon and southwest Washington State), (2) Kaiser Permanente Hawaii, (3) Kaiser Permanente Northern California, and several health clinics in the OCHIN, Inc. community health center network (federally qualified health centers) from Oregon and Montana were eligible for inclusion in this study.

We conducted a two-level eligibility screening process. Children who fit the inclusion criteria were first identified through the electronic health record (EHR), and eligibility was subsequently confirmed by the parent/guardian during the study enrollment process. The Kaiser Permanente Northwest Institutional Review Board (IRB), the single IRB for all collaborating sites, approved this study.

#### Inclusion

Using the EHR, we identified all children between the ages of 3 and 16.5 years with at least one face-to-face encounter (inpatient or outpatient ambulatory visit) during a 2-year rolling lookback period. Clinical diagnoses made at greater than one year of age were used to determine study eligibility. To be eligible for the ASD group the child must have met one of the following criteria: 1) two or more diagnoses of ASD (ICD-9 code 299.0, 299.8; ICD-10 codes F84.0, F84.5, F84.8) separated by > 30 days in the EHR; 2) an active ASD diagnosis on the EHR “ongoing problem list”; 3) one diagnosis of ASD from a specialty ASD clinic/provider identified by each site separately. Children in the asthma group were required to have two or more encounter-based, or medication-based diagnoses of asthma (ICD-9 code 493.0, 493.1, 493.9; ICD-10 code J45.2, J45.3, J45.4, J45.5, J45.90, J45.99) in the lookback period, separated by > 30 days. Children who fit the criteria for both ASD and asthma were eligible to participate and were included as part of the ASD group. Children with no diagnoses in the EHR of ASD at any time, or asthma during the lookback period, were eligible for the control group.

#### Exclusion

Children who were on each site’s “Do-Not-Contact for research” list, children with a cancer diagnosis in the EHR within the previous 36 months, and children who had died were excluded from the study population. Families were ineligible at screening if the respondent was a foster parent or if the child did not live with the parent at least 50% of the time, or if the respondent reported that the child had cancer within the previous 36 months. In addition, we compared the respondent report of their child’s health condition to the group assigned by the EHR criteria, and the child was excluded if the respondent-reported and EHR-assigned health groups did not match.

### Recruitment and survey administration

Approximately once a month over the 16-month recruitment period (November 2017 through February 2019), each study site extracted a random sample based on current recruitment capacity from the eligible population; children from racial and/or ethnic minority groups were oversampled. Because families were eligible based on the child’s diagnosis, a letter, addressed to “The Parent/Guardian of [child full name]”, was sent to the mailing address in the child’s EHR. Eligible children in the asthma and control groups were selected to match to the age and sex distribution of the eligible ASD group selected for recruitment.

The letter introduced the study and provided the study website and a child-specific enrollment code. Starting approximately one week after the mailing, trained recruiters attempted to contact parents/guardians by phone who had not yet responded. Up to three recruitment phone calls were made, each separated by at least 5 days. At one site, 1–2 emails to the child’s parent/guardian were also sent, and at another site every family that had not responded and that still met the inclusion/exclusion criteria was mailed a final invitation letter approximately 4 weeks prior to the study enrollment close date.

Surveys were programmed in REDCap [24, 25] (Research Electronic Data Capture). Once enrolled in the study, participants could complete the survey online or over the phone with an interviewer. All recruitment and survey materials, including recruitment phone calls, were offered in English and Spanish.

To capture a complete picture of a full year of family data while reducing the cognitive burden required for long recall periods and recall bias, participants were asked to complete the survey 3 times, 4 months apart. At each survey, the participant was asked to report on the previous 4 months. The first survey was completed at enrollment. For the subsequent two surveys, participants were emailed a notification at the email address they had provided in the first survey or contacted by phone if they did not provide an email address. Up to two reminder emails were sent, approximately one week apart, to non-responders for Surveys 2 and 3. Incentives in the form of gift cards were provided for participation: $30 for the first survey, $40 for the second, $50 for the third. Participants who completed all three surveys received an additional $15.

### Survey content

The r-Kids survey consists of child-, parent-, and household-focused assessments. (Table 1 provides a brief description of each measure along with the primary computed scales.) The key measure is the Family Economic Impact Inventory (FEII), which is a prospective measure of detailed family costs from multiple domains [26]. The FEII covers financial and time costs and employment impacts, as well as the overall financial impact of caring for a child’s health. It takes a broader view than most surveys of financial costs and includes typical costs such as copayments as well as less commonly measured costs such as payments for condition-related services outside the health care system. The FEII also includes comprehensive measurement of time costs, which covers items such as the time to arrange for health care services, take children to such services, and to manage the administrative burden of coordinating with insurance companies, schools, and others related to a child’s health. Employment impacts include both the impact of a child’s health on the decision to work, and hours worked, as well as the impact of the child’s health condition on missed time from work and productivity while at work. Finally, the FEII includes items to measure the overall financial impact of a child’s health on family economic well-being. These items include assessments of financial hardship such as inability to pay for care and incurring debt to pay for care [26]. Two types of psychometric analyses were conducted when the measure was developed: test–retest analyses 1 week apart, and a comparison of the parent-reported responses to medical records [27]. (See supplementary material for the FEII measure.)

**Table 1:** List of survey measures with descriptive details and method of administration

Measure	Description	Key domains/scales	Administration
**Family Economic Impact Inventory (FEII) **[26, 27]	Financial, time, and employment costs across different life domains. Utilization and cost are both captured, when appropriate to the domain. Questions are framed to remind respondent to report focused on the child’s health care needs specifically	Mental health, medical, and other services. Includes medication and medical equipment School services related to the child’s health Childcare services related to the child’s health Home and informal health related activities related to the child’s health Employment Family finances	All time points. All health groups
**Workplace Flexibility Scale **[28]	Employer policies related to workplace flexibility	Work flexibility benefits offered Work flexibility benefits used	All time points. All health groups
**Pediatric Quality of Life Inventory (PedsQL) **[29]	Child quality of life	Emotional functioning Social functioning School functioning Physical functioning	Baseline and Survey 3. All health groups
**Strengths and Difficulties Questionnaire (SDQ) **[30]	Child behavioral problems	Emotional problems Conduct problems Hyperactivity Peer problems Prosocial Impact on the family of child behavioral problems score	Baseline only. All health groups
**Social Responsiveness Scale (SRS) **[31]	ASD severity	A single ASD severity score	Baseline only. ASD only
**PROMIS Asthma Impact 8a (PROMIS-Asthma) **[32]	Asthma severity	A single asthma severity score	All time points. Asthma only
**Parenting Stress Scale (PSS) **[33]	Parental stress	Positive parenting experience score Negative parenting experience score	Baseline only. All health groups
**Caregiver Strain Questionnaire (CSQ) **[34]	Parent/caregiver strain. Designed for families with members that have mental health problems	Objective caregiver strain Subjective caregiver strain	Baseline and Survey 3 ASD only
**Demographic and descriptive characteristics**	Child, parent, family, and household characteristics		Baseline, Survey 3 All health groups

Existing validated instruments measuring the child’s quality of life (Pediatric Quality of Life Inventory; PedsQL [29]) and behavior (Strengths and Difficulties Questionnaire [30]); as well as symptom severity for children in the ASD (Social Responsiveness Scale [31]) and asthma (PROMIS Asthma Impact 8a [32]) groups, were also included. We assessed parenting stress (Parenting Stress Scale [33]), caregiver strain (Caregiver Strain Questionnaire [34]; ASD only), and parent physical and mental health in addition to collecting detailed family and household demographic variables.

### Analytic approach

We conducted descriptive analyses of analytic variables to ensure data quality, and to confirm distributions were in plausible ranges and were consistent with expected distributions. We further reviewed details of extreme or inconsistent observations to rule out data entry errors. To describe the enrolled sample overall, and by health group, Ns (percentages) are reported in tables, and chi-square tests were used for comparisons. To examine differences in the demographic, quality of life, and health characteristics of the enrolled families by health group, we used multivariable regression, controlling for study site and the child’s age and gender. Parameter estimates of health group were used to compare the ASD group to the asthma and control group separately as well as to compare the asthma group to the control group. We used a two-sided α = 0.05 for inference, and all data management and analyses were conducted using SAS version 9.4 and Stata version 16.1.

## Results

### Recruitment and retention

We mailed letters to 6,845 families. Of those, 277 letters were returned as undeliverable by the post office. Thirty-five families were determined to be ineligible when contacted by phone, resulting in 6,533 potentially eligible families. Of those, 1,707 consented to participate (response rate = 26.1%). Upon further screening, 246 of the consented families were ineligible, resulting in 1,461 families that enrolled in the study.

Of the 1,461 families that enrolled (564 ASD, 468 asthma, 429 control), 290 (19.9%) only participated in the first survey, 188 (12.9%) only participated in two surveys, and 983 (67.3%) participated in all three surveys.

### Participation bias

We compared participating families (*N* = 1,461) to families that met our eligibility criteria and that we attempted to recruit but that chose not to participate (*N* = 4,826) on the child characteristics that were available through the EHR (i.e., health group, age group, gender, race/ethnicity, and indication of public insurance in two years prior to sample date; Table 2). Of the recruited families, 38.6% in the ASD group participated compared to 32% in the asthma group and 29.4% in the control group (*p* < 0.001). Non-Hispanic white families constituted 43.8% of the participants but only 37.2% of the non-participants (*p* < 0.001). In addition, we found that families with public health insurance were less likely to participate than families without it (20.6% vs 26.3%, respectively; *p* < 0.001). There were no differences in study participation based on child gender or age.

**Table 2:** Characteristics of respondents and eligible non-respondents

	**Declined or no response**^**a**^		**Consented and eligible**^**b**^		***p*-value**
	**N**	**%**	**N**	**%**	
**Number of people sampled**	**4,826**		**1,461**		
Health Group					< 0.001
ASD	1,458	30.2	564	38.6	
Asthma	1,676	34.7	468	32.0	
Control	1,692	35.1	429	29.4	
Gender male (yes)	3,838	79.5	1,152	78.9	0.575
Race/ethnicity					< 0.001
White (non-Hispanic)	1,794	37.2	640	43.8	
Hispanic	947	19.6	246	16.8	
Native Hawaiian or other Pacific Islander	445	9.2	113	7.7	
Asian	692	14.3	172	11.8	
Black or African American	309	6.4	86	5.9	
Other or multiple race/ethnicity	232	4.8	90	6.2	
Unknown	407	8.4	114	7.8	
Age^c^					0.060
Early childhood (3–5 years)	1,009	20.9	337	23.1	
Middle childhood (6–11 years)	2,177	45.1	670	45.9	
Adolescent (12–17 years)	1,640	34.0	454	31.1	
Indication of public insurance in 2 years prior to sample date (yes)	1,267	26.3	301	20.6	< 0.001

Chi-square tests were used to examine differences in the distribution between respondents and eligible non-respondents for each characteristic

^a^Ineligible families were not included in this group

^b^Data for this table was extracted from the EHR only. We chose not to use self-reported data for the respondents so the data source for both groups was the same

^c^Age group for both groups is based on the date the sample was pulled

### Descriptive characteristics

The study children were predominantly male (79%), were well represented in each age group (3–5 years = 22.3%, 6–11 years = 45.6%, 12–17 years = 32.1%), and were a racially and ethnically diverse group (Table 3). There were no differences across health groups in the gender or age distribution of the children. Children in the ASD and control groups were more likely to be non-Hispanic white (44.3% and 45.9% respectively) compared to asthma group (38.8%; *p* = 0.002). Public insurance was significantly more common for children in the ASD (31%) and asthma groups (29%) compared to control group children (16%) while children in the control group were more likely to be uninsured than children in the other groups (*p* < 0.001).

**Table 3:** Child characteristics reported at the first survey by health group

	**Overall**	**ASD**	**Asthma**	**Control**	***p*-value**
	**N (%)**	**N (%)**	**N (%)**	**N (%)**	
**Consented and eligible**	**1461**	**564**	**468**	**429**	
Gender: Male (yes)	1152 (78.9)	430 (76.2)	375 (80.1)	347 (80.9)	0.148
Age^a^					0.176
Early childhood (3–5 years)	326 (22.3)	128 (22.7)	90 (19.2)	108 (25.2)	
Middle childhood (6–11 years)	666 (45.6)	246 (43.6)	224 (47.9)	196 (45.7)	
Adolescent (12–17 years)	469 (32.1)	190 (33.7)	154 (32.9)	125 (29.1)	
Race/Ethnicity^b^ (*n* = 1444)					0.002
White (non-Hispanic)	621 (43)	245 (44.3)	181 (38.8)	195 (45.9)	
Hispanic	313 (21.7)	134 (24.2)	101 (21.7)	78 (18.4)	
Native Hawaiian or Other Pacific Islander	128 (8.9)	28 (5.1)	58 (12.4)	42 (9.9)	
Asian	141 (9.8)	55 (9.9)	47 (10.1)	39 (9.2)	
Black or African American	57 (3.9)	22 (4)	24 (5.2)	11 (2.6)	
Other or multiple race/ethnicity	184 (12.7)	69 (12.5)	55 (11.8)	60 (14.1)	
Insurance type (*N* = 1307)					< 0.001
Private	883 (67.6)	322 (65.4)	273 (64.8)	288 (73.1)	
Public	340 (26)	154 (31.3)	123 (29.2)	63 (16)	
Uninsured	84 (6.4)	16 (3.3)	25 (5.9)	43 (10.9)	

Data for this table was preferentially from the respondent-reported survey responses. For 187 children, missing race/ethnicity data was supplemented from the EHR data. For gender and age, the percentages presented are based on *N* = 1461

Chi-square tests were used to examine differences in the distribution between health groups (ASD, Asthma, Control) for each characteristic

^a^Age group is based on child age the date the baseline survey was completed

^b^Race/ethnicity was assigned as follows: If Hispanic ethnicity was reported, the child was categorized as Hispanic. For the remaining children, if Native Hawaiian or Other Pacific Islander was chosen, the child was categorized as Native Hawaiian or Other Pacific Islander. The next categorization was of more than one race/ethnicity. The remaining uncategorized children were categorized as White, Black, or Asian

The majority of survey respondents were female (86%), had a college degree (62%), and were married or living with a partner (79%; Table 4). ASD group respondents were less likely to be employed (73%) than those in the asthma or control groups (both 80%; *p* = 0.023). Household income and insurance type varied significantly by group as well. Only 32% of the control group reported a household income ≤ $4,000/month compared to 41% of asthma and 38% of ASD families (*p* = 0.006). There were no group differences in household size; however, families in the ASD (43%) and asthma groups (47%) were significantly more likely to have other children in the household with a serious health condition compared to control families (22%, *p* < 0.001).

**Table 4:** Survey respondent and household characteristics at baseline by health group

	**Overall**	**ASD**	**Asthma**	**Control**	***p*-value**
	**N (%)**	**N (%)**	**N (%)**	**N (%)**	
**Respondent characteristics**					
Gender: Female (*N* = 1263)	1086 (86)	398 (85.2)	360 (88)	328 (84.8)	0.341
Biological parent (*N* = 1263)	1207 (95.6)	438 (93.8)	393 (96.1)	376 (97.2)	0.048
Education level (*N* = 1253)					0.555
High school or less	171 (13.6)	56 (12.1)	62 (15.3)	53 (13.7)	
Some college	301 (24)	116 (25.1)	101 (25)	84 (21.8)	
College degree	436 (34.8)	170 (36.7)	131 (32.4)	135 (35)	
Graduate degree	345 (27.5)	121 (26.1)	110 (27.2)	114 (29.5)	
Married or living with partner (*N* = 1259)	988 (78.5)	375 (80.6)	311 (76.2)	302 (78.2)	0.282
Respondent employed (*N* = 1290)	995 (77.1)	354 (73)	331 (79.6)	310 (79.7)	0.023
**Household characteristics**					
Household monthly income (*N* = 1218)					0.006
≤ $4,000	448 (36.8)	169 (37.5)	163 (40.8)	116 (31.6)	
$4,001-$8,000	430 (35.3)	172 (38.1)	134 (33.5)	124 (33.8)	
> $8,000	340 (27.9)	110 (24.4)	103 (25.8)	127 (34.6)	
Number of adults in the household (*N* = 1249)					.265
1	182 (14.6)	64 (13.8)	60 (14.9)	58 (15.1)	
2	828 (66.3)	323 (69.8)	264 (65.5)	241 (62.9)	
More than 2	239 (19.1)	76 (16.4)	79 (19.6)	84 (21.9)	
Number of children in the household (*N* = 1254)					.357
1	334 (26.6)	117 (25.3)	107 (26.3)	110 (28.6)	
2	565 (45.1)	213 (46.1)	173 (42.5)	179 (46.5)	
3	226 (18)	89 (19.3)	75 (18.4)	62 (16.1)	
More than 3	129 (10.3)	43 (9.3)	52 (12.8)	34 (8.8)	
Other children in the household with a serious health condition (*N* = 920)	348 (37.8)	147 (42.6)	141 (47)	60 (21.8)	< 0.001

Chi-square tests were used to examine differences in the distribution between health groups (ASD, Asthma, Control) for each characteristic

### Mental health, physical health, and quality of life

Overall, children in the ASD group had significantly lower quality of life and higher behavioral difficulties scores compared to children in the asthma or control groups (Table 5). Children in the ASD group had a significantly lower PedsQL psychosocial health score (M = 55.9) compared to the asthma and control groups (M = 77.2 and M = 79.0 respectively, ps < 0.001). The PedsQL physical health summary score was also significantly lower in the ASD group (M = 71.7) compared to the asthma and control groups (M = 82.3 and M = 88.0 respectively, ps < 0.001). Children in the ASD group also had significantly higher levels of internalizing and externalizing problems/symptoms, and significantly lower prosocial behavior scores compared to children in the other groups (ps < 0.001, see Table 5). Children in the asthma group had higher SDQ internalizing scores and lower PedsQL physical health scores than children in the control group (*p* = 0.033 and *p* < 0.001, respectively).

**Table 5:** Multivariable regression examining child and survey respondent mental health, physical health, and quality of life by health group

				**Adjusted *p*-values**^**a**^			
	**ASD **	**Asthma **	**Control **	**Overall model**	**ASD vs. control**	**ASD vs. Asthma**	**Asthma vs. Control**
	**(*N*** **= 564)**	**(*N*** **= 468)**	**(*N*** **= 429)**				
	**M (SD)**	**M (SD)**	**M (SD)**				
**Child characteristics**							
Pediatric Quality of Life (PedsQL)							
Psychosocial Health Summary score (*N* = 1,425)	55.9 (15.9)	77.2 (15.9)	79.0 (15.0)	< .001	< .001	< .001	.177
Physical Health Summary score (*N* = 1,430)	71.7 (21.7)	82.3 (19.0)	88.0 (16.2)	< .001	< .001	< .001	< .001
Strengths and Difficulties Questionnaire (SDQ; *N* = 1,267))							
Externalizing score	8.5 (3.9)	3.8 (3.3)	3.6 (3.3)	< .001	< .001	< .001	.602
Internalizing score	9.6 (3.8)	5.6 (3.9)	5.1 (3.8)	< .001	< .001	< .001	.033
Prosocial score	5.3 (2.5)	8.3 (1.8)	8.2 (1.9)	< .001	< .001	< .001	.664
**Survey respondent characteristics**							
Parenting stress score (*N* = 1,151)	40.8 (10.4)	34.2 (8.7)	34.7 (9.2)	< .001	< .001	< .001	.613
Respondent overall physical health (*N* = 1,257)	3.3 (1.0)	3.3 (0.9)	3.5 (0.9)	.020	.015	.936	.014
Respondent overall mental health (*N* = 1,249)	3.5 (1.1)	3.6 (0.9)	3.7 (1.0)	.002	< .001	.057	.111

Raw means and standard deviations

The *p*-value for the Overall Model reports the omnibus test of significance

^a^*p*-values adjusted for age, gender, and study site

Survey respondents for children in the ASD group reported significantly higher levels of parenting stress than respondents from the other groups (both comparisons *p* < 0.001). While respondents in the ASD group reported significantly worse mental (*p* < 0.001) and physical (*p* = 0.015) health than respondents in the control group, there were no significant differences when compared with the asthma group. Respondents with children in the asthma group reported significantly worse physical health compared to respondents in the control group (*p* = 0.014). See supplemental Table 1 for the multivariable regression analysis parameters estimates and standard errors.

## Discussion

The r-Kids study successfully recruited a large and ethnically and geographically diverse sample of families of children with ASD, asthma, and a control group of families with children having neither health condition. It is one of the first studies to collect comprehensive data on the economic burden to families of caring for children’s health. We compared the characteristics of enrolled families in each health group and found that children with ASD scored significantly worse on all measures of quality of life and physical, behavioral, and socioemotional adjustment compared to the other groups. In addition, parent/guardian respondents with children with ASD reported worse physical and mental health and more parenting stress than respondents with children in the control group. These findings are consistent with previous studies of children with ASD and their parents [35].

The r-Kids study focused on advancing the methods of collecting data on family economic burden. Data were collected using an instrument specifically designed to measure family costs that provides a more nuanced picture of the many types of costs families experience. Data were collected at 4-month intervals to improve the accuracy of reported costs. Most prior studies have used 12-month recall periods which makes it more challenging for parents to provide accurate estimates of costs, particularly frequently occurring costs such as time spent caregiving [7, 8, 16]. By conducting the survey at three different time points over a 12-month period we ensured that costs which may differ by season (e.g., school related costs) were captured while still gathering data that will allow direct comparison of one-year costs across studies [13, 15, 16, 18].

Rather than relying solely on parent report, we used two independent sources of information to determine the child’s health group. Children were identified using clinical diagnoses from health system EHRs, and the health condition was independently confirmed by the parent/guardian respondent. Most previous studies have relied solely on parent report of the child’s health condition, which could lead to misclassification of children.

The r-Kids study presents several additional advantages over prior estimates of the economic burden to families of caring for their child’s health. The sample is relatively large with 1,461 families enrolled, which included 564 families of children and youth with ASD. Compared to most previous studies, which rely on claims from commercially insured children or Medicaid claims only, we included children from a broad economic spectrum including children who were insured through their parent’s employment, who were publicly insured, and children without insurance. In addition, more than half the participants were from racial and ethnic groups who are historically underserved and under-researched in the United States [36]. Both English and Spanish speaking families could participate in their native language, which is uncommon in prior studies of family cost. The diversity of participants in this study will allow for important analyses of racial and ethnic disparities in the economic impacts of caring for child health. The sample also included a wide age range with robust sample sizes of children from early childhood, middle childhood, and adolescence, which will allow comparisons of costs at different developmental stages.

Most previous studies that focused on ASD have not included a comparison group or relied on only one related comparison group (e.g., children with intellectual disabilities). We wanted to understand the incremental impact from a broader perspective, so we chose to include a comparison group with a common chronic physical health condition, asthma, and a group of children from the general pediatric population.

Most studies of the cost of ASD and other health conditions (e.g., cancer) have focused on costs to families related to the health care system, such as co-payments for services [3, 4, 17]. Yet many costs incurred by families happen outside of the health care system. Few studies have focused primarily on family costs, and those that have did not consider the broad array of costs that families might experience, such as costs of time spent in managing a child’s health and health care needs. Our study addresses these limitations by focusing on a broad array of costs that families incur.

The r-Kids study has several limitations. The study recruited participants from health care systems in five different states, but the sample may not be generalizable beyond these populations. The vast majority of children had health insurance, recent engagement with a usual source of care, and were reachable by phone and/or email, which may have excluded some families without these resources. The nature of the electronic survey may have also selected for somewhat more educated participants with better access to electronics. Our response rate of 26% is reasonable for an online survey format [37–39]; however, r-Kids participants may have differed from the underlying population from which the sample was recruited. We explored this with available data and found some differences between respondents and non-respondents, but the differences were small. Finally, we used an observational design and therefore cannot demonstrate causation. In addition, we have limited data on ASD phenotype beyond symptom severity, such as IQ and language level.

## Conclusions

With more than 1400 families from diverse sociodemographic backgrounds participating in the r-Kids study, and the use of the FEII, we collected comprehensive data on the economic burdens to families associated with caring for a child’s health. In future publications that will report empirical estimates of each outcome in our conceptual framework, we hope to provide much-needed context by comparing the incremental economic cost differences for families caring for a child with a CHC above and beyond caring for a child without these types of conditions. The analysis of employment impacts for families of children with ASD compared to families of children with asthma and families of children with neither condition has been published [40]. Future reports will examine the financial costs, time costs, the impact of employer policies, as well as examine the role of race and ethnicity, on the economic burden experienced by families. The direct experience of the family is a critical and often neglected perspective. Improving our understanding of the economic impact of different child chronic health conditions on families is crucial to the development of more effective and equitable policies.

## Supplementary Information

**Additional file 1**.

**Additional file 2:** **SupplementalTable 1. **Multivariable Regression Analysis Parameters Estimates (PE) andStandard Errors (SE) of Child and Survey Respondent Mental Health, PhysicalHealth, and Quality of Life.

## Data Availability

The datasets generated and/or analyzed during the current study are available in the National Database for Autism Research (NDAR) repository, https://nda.nih.gov/edit_collection.html?id=2468.
