# Supplementary material for: A framework for measuring the cost to families of caring for children’s health: the design, methodology, and study population of the r-Kids study
Source: BMC Pediatr. 2023 Mar 20;23:128. doi: 10.1186/s12887-023-03893-7 (PMC10025806; doi:10.1186/s12887-023-03893-7)
Supplement: Supplementary file 1 — Additional file 1. [file 12887_2023_3893_MOESM1_ESM.docx]

**Introduction**

The Family Economic Impact Inventory (FEII) was developed through a grant from the National Institute of Mental Health to comprehensively measure economic impacts of caring for a child with emotional, behavioral, or developmental conditions. The aim of the FEII is to examine a broad range of economic impacts beyond monetary costs, such as copayments for medical services, that have been studied more extensively. The rationale for this framework is that economic impacts extend beyond monetary costs and include time families spend related to their child’s health, and changes that families make to accommodate the needs of their child, such as changes to employment, and financial choices (e.g., using savings to pay for care) that may have long-term economic impacts on families.

The initial work in the development of the FEII included qualitative interviews with parents of children with emotional, behavioral, and/or developmental conditions to identify ways in which family economic well-being was impacted when caring for a child with one or more of these conditions. The first version of the FEII was then piloted with families followed by cognitive interviews to help to refine the questions.[1] We have conducted two types of psychometric analyses as we developed the instrument. First, we conducted test-retest analyses 1 week apart, and second, we compared parts of the parent-reported information in the FEII to medical records.[2]

During the development of the FEII we identified six domains in which families reported economic impact: childcare, school, medical and other services, at-home care, family finances, and parent employment.

**Administration of the FEII in the r-Kids study**

The purpose of the r-Kids study was to compare the economic impact of caring for a child with autism spectrum disorders (ASD), another chronic health condition (asthma), and neither health condition (control).

Below are the core questions from the FEII. The FEII was completed using a REDCap survey, either by the parent participant or via a phone interview if preferred by the parent. Guidance and instructions visible to the respondent are in ***bold italics***. Notes regarding response options are provided in *italics*.

Some notes about the administration:

- The REDCap version of the survey contains extensive skip patterns which are not noted here. Follow-up questions were only shown to parents whose answer to the initial question triggered the follow-up.
- Brackets in the questions note when content was included that varied based on the REDCap survey programming (e.g. [name]).
- As appropriate, REDCap validation features were utilized to reduce accidental mis-entry in open-text (e.g., where a number or dollar amount was requested).
- Where appropriate, questions in the survey include the response options “Don’t know” and “Prefer not to answer.”
- Throughout the survey questions with response options such as “Other” or “More than five days” were followed by an open-response text box.

**Section 1. Childcare**

| **Question** | **Response options** |
| --- | --- |
| ***The following questions are about the extra time or costs for childcare your family may have because of [name]'s health. This could be at a preschool program, an afterschool program, or another childcare arrangement that occurs regularly (at least once per week during most weeks).*** |  |
| ***The period of time you will be answering about is the 4-month period beginning around [date calculated by REDCap].*** |  |
| During the LAST 4 MONTHS, was [name] regularly cared for by someone other than you or another person in your household? | Yes/No  *[If response is “No”, the follow up questions in this section are skipped]* |
| During the LAST 4 MONTHS, what type of childcare did [name] regularly receive? |  |
| A relative in your home | Yes/No |
| Care in a relative's home | Yes/No |
| Non-relative in your home (babysitter or nanny) | Yes/No |
| Head start or Early Intervention program | Yes/No |
| Childcare center | Yes/No |
| Preschool or nursery school | Yes/No |
| Before or after care program at school | Yes/No |
| Special childcare program for children with special health care or developmental needs | Yes/No |
| Other | Yes/No |
| During the LAST 4 MONTHS did your family pay any money for childcare or preschool because of [name]'s health or chronic health condition? (For example, extra tuition or special fees) | Yes/No |
| Altogether, during the LAST 4 MONTHS, how much did your family pay for childcare because of your child's health or chronic health condition? | *Open-ended response* |
| During the LAST 4 MONTHS, did you ever have to pick up [name] from childcare unexpectedly due to their health or chronic health condition? | Yes/No |
| Altogether, in the LAST 4 MONTHS, about how many times did you unexpectedly pick up [name] from childcare because of their health or chronic health condition? | 1 time; 2 times; 3 times; 4 times; 5 times; More than 5 times |
| During the LAST 4 MONTHS, did you ever have to miss work when picking [name] up unexpectedly from childcare because of their health or chronic health condition? | Yes/No |
| Altogether, during the LAST 4 MONTHS, about how many hours did you miss from work when picking up [name] unexpectedly from childcare because of their health or chronic health condition? | Less than 1 hour; 1 hour; 2 hours; 3 hours; 4 hours; 5 hours; More than 5 hours |
| During the LAST 4 MONTHS, has [name] missed whole days of childcare due to their health or chronic health condition? | Yes/No |
| Altogether, during the LAST 4 MONTHS, about how many times did [name] miss whole days of childcare due to their health or chronic health condition? | 1 time; 2 times; 3 times; 4 times; 5 times; More than 5 times |
| During the LAST 4 MONTHS did you ever have to miss work due to [name] missing whole days of childcare because of their health or chronic health condition? | Yes/No |
| Altogether, during the LAST 4 MONTHS, about how many days did you miss from work due to [name] missing whole days of childcare related to their health or chronic health condition? | Less than 1 day; 1 day; 2 days; 3 days; 4 days; 5 days; More than 5 days |
| During the LAST 4 MONTHS, has [name] received any medical care, individual counseling or evaluation from a nurse, counselor, psychologist or other medical provider at any childcare program, related to [name]'s health or chronic health condition? | Yes/No |
| During the LAST 4 MONTHS did you attend any of these medical, counseling or evaluation appointments? | Yes/No |
| Altogether, during the LAST 4 MONTHS, how many times did you attend appointments for medical, individual counseling or evaluation from the childcare program related to [name]'s health or chronic health condition? | 1 time; 2 times; 3 times; 4 times; 5 times; More than 5 times |
| During the LAST 4 MONTHS did you ever have to miss work to attend individual counseling or evaluation appointments from the childcare program related to [name]'s health or chronic health condition? | Yes/No |
| Altogether, during the LAST 4 MONTHS, about how many hours did you miss from work while attending these appointments from the childcare program related to [name]'s health or chronic health condition? | Less than 1 hour; 1 hour; 2 hours; 3 hours; 4 hours; 5 hours; More than 5 hours |
| During the LAST 4 MONTHS, did your family have to pay for any of these services from the childcare program related to [name]'s health or chronic health condition? | Yes/No |
| Altogether, during the LAST 4 MONTHS, about how much did your family pay for these services from the childcare program related to [name]'s health or chronic health condition? | *Open-ended response* |
| During the LAST 4 MONTHS, did you attend any other meetings at a childcare program related to [name]'s health or chronic health condition? | Yes/No |
| Altogether, in the LAST 4 MONTHS, how many times did you attend any of these other meetings related to [name]'s health or chronic health condition? | 1 time; 2 times; 3 times; 4 times; 5 times; More than 5 times |
| During the LAST 4 MONTHS did you ever miss work to attend other meetings at the childcare program related to [name]'s health or chronic health condition? | Yes/No |
| Altogether, during the LAST 4 MONTHS, about how many hours did you miss from work while attending other meetings at the childcare program related to [name]'s health or chronic health condition? | Less than 1 hour; 1 hour; 2 hours; 3 hours; 4 hours; 5 hours; More than 5 hours |
| During the LAST 4 MONTHS, were you unable to find a childcare program due to [name]'s health or chronic health condition? | Yes/No |
| During the LAST 4 MONTHS, did you ever lose childcare or have to stop childcare arrangements due to [name]'s health or chronic health condition? | Yes/No |

**Section 2. School**

| **Question** | **Response options** |
| --- | --- |
| ***The next questions are about extra time or costs for school your family may have because of [name]'s health or chronic health conditions.*** |  |
| At any time during the LAST 4 MONTHS has [name] attended school (kindergarten to high school)? | Yes/No  *[The response of “Yes” or “No” determines which questions below the respondent receives]* |
| Why didn't [name] attend school at all during the LAST 4 MONTHS? |  |
| Child isn't old enough to start school yet | Yes/No |
| School was out of session during this time | Yes/No |
| Child was not able to attend school due to a health or chronic health condition | Yes/No |
| Child graduated from high school | Yes/No |
| Another reason | Yes/No |
| What type of school did [name] attend in the LAST 4 MONTHS? (Please choose the best fit) | Public School; Private school; Home school; Another type of school |
| During the LAST 4 MONTHS did your family pay any extra money for this school because of [name]'s health or chronic health condition? (For example, extra tuition or special fees) | Yes/No |
| Altogether, during the LAST 4 MONTHS, how much did your family pay for this school because of your child's health or chronic health condition? | *Open-ended response* |
| During the LAST 4 MONTHS did your family spend any money on home schooling because of [name]'s health or chronic health condition? | Yes/No |
| Altogether, during the LAST 4 MONTHS, how much did your family pay for home schooling because of your child's health or chronic health condition? | *Open-ended response* |
| During the LAST 4 MONTHS, did you ever have to pick up [name] from school unexpectedly because of their health or chronic health condition? | Yes/No |
| Altogether, during the LAST 4 MONTHS, about how many times did you unexpectedly pick up [name] from school because of their health or chronic health condition? | 1 time; 2 times; 3 times; 4 times; 5 times; More than 5 times |
| During the LAST 4 MONTHS, did you ever miss work when picking up [name] unexpectedly because of their health or chronic health condition? | Yes/No |
| Altogether, during the LAST 4 MONTHS, about how many hours did you miss from work when picking up [name] because of their health or chronic health condition? | Less than 1 hour; 1 hour; 2 hours; 3 hours; 4 hours; 5 hours; More than 5 hours |
| During the LAST 4 MONTHS, has [name] missed whole days of school because of their health or chronic health condition? | Yes/No |
| Altogether, during the LAST 4 MONTHS, about how many times did [name] miss whole days of school because of their health or chronic health condition? | 1 time; 2 times; 3 times; 4 times; 5 times; More than 5 times |
| During the LAST 4 MONTHS did you ever have to miss work due to [name] missing whole days of school because of their health or chronic health condition? | Yes/No |
| Altogether, during the LAST 4 MONTHS, about how many days did you miss from work due to [name] missing whole days of school related to their health or chronic health condition | Less than 1 day; 1 day; 2 days; 3 days; 4 days; 5 days; More than 5 days |
| During the LAST 4 MONTHS, have you spent time communicating or coordinating with teachers or other school staff about [name]'s health or chronic health condition? For example, talking to teachers about [name]'s medications or coordinating with teachers about missed assignments due to school absences. | Yes/No |
| Altogether, during the LAST 4 MONTHS, about how many hours did you spend communicating or coordinating with [name]'s teachers or school about their health or chronic health condition? | Less than 1 hour; 1 hour; 2 hours; 3 hours; 4 hours; 5 hours; More than 5 hours |
| During the LAST 4 MONTHS did you ever have to miss work due to spending time communicating or coordinating with [name]'s teachers or school about their health or chronic health conditions? | Yes/No |
| Altogether, during the LAST 4 MONTHS, about how many hours did you miss from work due to communicating or coordinating with [name]'s teachers or school about their health or chronic health conditions? | Less than 1 hour; 1 hour; 2 hours; 3 hours; 4 hours; 5 hours; More than 5 hours |
| During the LAST 4 MONTHS, have you spent time volunteering in the classroom or school related to [name]'s health or chronic health condition? | Yes/No |
| Altogether, during the LAST 4 MONTHS, about how many hours did you spend volunteering in the classroom or school related to [name]'s health or chronic health condition? | Less than 1 hour; 1 hour; 2 hours; 3 hours; 4 hours; 5 hours; More than 5 hours |
| During the LAST 4 MONTHS did you ever have to miss work due to volunteering in [name] classroom or school because of their health or chronic health condition? | Yes/No |
| Altogether, during the LAST 4 MONTHS, about how many hours did you miss from work due to volunteering in [name]'s classroom or school because of their health or chronic health condition? | Less than 1 hour; 1 hour; 2 hours; 3 hours; 4 hours; 5 hours; More than 5 hours |
| During the LAST 4 MONTHS, have you spent extra time helping [name] with school work outside of school time because of their health or chronic health condition? (such as helping them catch up after missing school due to appointments) | Yes/No |
| During the LAST 4 MONTHS, about how many hours did you spend in a typical week helping [name] with school work due to their health or chronic health condition? | Less than 1 hour; 1 hour; 2 hours; 3 hours; 4 hours; 5 hours; More than 5 hours |
| During the LAST 4 MONTHS, has [name] received medical care, individual counseling or evaluation from a counselor, psychologist, or other medical provider from the school or education system related to their health or chronic health condition? | Yes/No |
| During the LAST 4 MONTHS, did you attend any of these appointments with the school or education system that were related to their health or chronic health condition? | Yes/No |
| Altogether, during the LAST 4 MONTHS, how many times did you have to attend appointments for medical care, individual counseling or evaluation from the school or education system related to [name]'s health or chronic health condition? | 1 time; 2 times; 3 times; 4 times; 5 times; More than 5 times |
| During the LAST 4 MONTHS did you ever have to miss work to attend these appointments from the school or education system related to [name]'s health or chronic health condition? | Yes/No |
| Altogether during the LAST 4 MONTHS, about how many hours did you miss from work while attending these appointments from the school or education system related to [name] health or chronic health condition? | Less than 1 hour; 1 hour; 2 hours; 3 hours; 4 hours; 5 hours; More than 5 hours |
| During the LAST 4 MONTHS, did your family have to pay for any of these services from the school or education system related to [name]'s health or chronic health condition? | Yes/No |
| Altogether during the LAST 4 MONTHS, about how much did your family pay for these services from the school or education system related to [name]'s health or chronic health condition? | *Open-ended response* |
| During the LAST 4 MONTHS, has [name] received special help in the classroom because of his or her health or chronic health condition? (such as a classroom aide to assist with classwork) | Yes/No |
| During the LAST 4 MONTHS, on a typical school day, how many hours does [name] receive special help in the classroom due to their health or chronic health condition? | Less than 1 hour; 1 hour; 2 hours; 3 hours; 4 hours; 5 hours; More than 5 hours |
| In the LAST 4 MONTHS, was [name] in a special education classroom due to their health or chronic health condition? | Yes/No |
| During the LAST 4 MONTHS, how often was [name] in a special education classroom due to their health or chronic health condition? | Every school day; Part of the school week; Other |

**Section 3. Medical care and other services**

| **Question** | **Response options** |
| --- | --- |
| ***The next questions are about services for [name]'s health or chronic health conditions. First, we will ask about medical care, next complementary or alternative care, then medicines. Remember, it’s OK to give your best guess. And the period of time you will be answering about is the 4-month period beginning around [date calculated by REDCap].*** |  |
| During the LAST 4 MONTHS, did [name] spend the night in a hospital or other facility? | Yes/No |
| Altogether, during the LAST 4 MONTHS, what was the total number of nights [name] stayed in a hospital or other facility?  If [name] stayed more than one time, please add all the nights the child was in a hospital or other facility in the LAST 4 MONTHS. | 1 night; 2 nights; 3 nights; 4 nights; 5 nights; More than 5 nights |
| What were the reasons for the time in the hospital or other inpatient facility? | ***Check all that apply.***  Injury or poisoning; Asthma; Other breathing problems (such as pneumonia or bronchitis); Concussion; Nervous system disorder (such as seizure); Autism spectrum disorders; Infections and parasitic diseases; Vomiting, diarrhea, severe stomach pain; Burns or other skin disorders; Mental health or behavioral problems; Broken bone, sprain; Substance use problems; Other reason |
| Altogether during the LAST 4 MONTHS, about how much did your family pay for these hospital or inpatient facility stays? | *Open-ended response* |
| During the LAST 4 MONTHS, has [name] had a visit to an emergency room or urgent care center? | Yes/No |
| Altogether, during the LAST 4 MONTHS, how many times did [name] have a visit to an emergency room or urgent care center? | 1 time; 2 times; 3 times; 4 times; 5 times; More than 5 times |
| What were the reasons for [name]'s visit(s) to an emergency room or urgent care center? | ***Check all that apply.***  Injury or poisoning; Asthma; Other breathing problems (such as pneumonia or bronchitis); Concussion; Nervous system disorder (such as seizure); Autism spectrum disorders; Infections and parasitic diseases; Vomiting, diarrhea, severe stomach pain; Burns or other skin disorders; Mental health or behavioral problems; Broken bone, sprain; Substance use problems; Other reason |
| Altogether during the LAST 4 MONTHS, about how much did your family pay for these emergency room or urgent care visits? | *Open-ended response* |
| During the LAST 4 MONTHS, has [name] had a visit to a primary care provider, including a pediatrician, nurse practitioner, physician's assistant or family medical doctor? | Yes/No |
| Altogether, during the LAST 4 MONTHS, how many times did [name] have a visit to a primary care provider? | 1 time; 2 times; 3 times; 4 times; 5 times; More than 5 times |
| What were the reasons for [name]'s visits to a primary care provider? | ***Check all that apply.***  Well-child visit, or check-up; Injury or poisoning; Asthma; Other breathing problems (such as pneumonia or bronchitis); Concussion; Nervous system disorder (such as seizure); Autism spectrum disorders; Infections and parasitic diseases; Vomiting, diarrhea, severe stomach pain; Burns or other skin disorders; Mental health or behavioral problems; Broken bone, sprain; Substance use problems; Other reason |
| Altogether during the LAST 4 MONTHS, about how much did your family pay for these visits to the primary care provider? | *Open-ended response* |
| During the LAST 4 MONTHS, has [name] had a visit to a medical specialist (such as neurologist, gastroenterologist, dermatologist) | Yes/No |
| Altogether, during the LAST 4 MONTHS, how many times did [name] have a visit to a medical specialist (such as a neurologist or gastroenterologist)?  Please add together all visits to different medical specialists, so if your child saw both a neurologist and a gastroenterologist, one time each, you would report 2 times. | 1 time; 2 times; 3 times; 4 times; 5 times; More than 5 times |
| What were the reasons for [name]'s visits to a medical specialist (such as a neurologist or gastroenterologist)? | ***Check all that apply.***  Skin problems; Diabetes; Asthma; Other breathing problems (such as pneumonia or bronchitis); Concussion; Nervous system disorder such as seizure); Autism spectrum disorders; Infections and parasitic diseases; Digestive or stomach problems; Mental health or behavior problems; Substance use problems; Cancer; Other reason |
| Altogether during the LAST 4 MONTHS, about how much did your family pay for visits to a medical specialist (such as a neurologist or gastroenterologist)? | *Open-ended response* |
| ***During the LAST 4 MONTHS, has [name] had a visit to any of the following health care providers?*** |  |
| Occupational therapist | Yes/No |
| Physical therapist | Yes/No |
| Speech therapist | Yes/No |
| Respiratory physiotherapist | Yes/No |
| Nutritionist | Yes/No |
| *For each health care provider marked “Yes” above, the following set of questions are presented* |  |
| Altogether, during the LAST 4 MONTHS, how many times did [name] have a visit to [health care provider]? | 1 time; 2 times; 3 times; 4 times; 5 times; More than 5 times |
| Altogether during the LAST 4 MONTHS, about how much did your family pay for visits to [health care provider]? | *Open-ended response* |
| During the LAST 4 MONTHS, has [name] had a visit to a mental health provider such as a psychologist, therapist, or counselor for any of the following mental health services? |  |
| Individual counseling or therapy | Yes/No |
| Group therapy | Yes/No |
| Case management | Yes/No |
| Other mental health service | Yes/No |
| *For each mental health service marked “Yes” above, the following questions are presented* |  |
| Altogether, during the LAST 4 MONTHS, how many times did [name] have a visit for [mental health service]?  Please do not include visits at school. We will ask about those separately. | 1 time; 2 times; 3 times; 4 times; 5 times; More than 5 times |
| Altogether during the LAST 4 MONTHS, about how much did your family pay for visits for [mental health service]? | *Open-ended response* |
| During the LAST 4 MONTHS, has [name] received any of the following services? |  |
| Applied behavioral analysis (ABA) | Yes/No |
| Asthma management group | Yes/No |
| Diabetes management group | Yes/No |
| Social skills training or group | Yes/No |
| Weight management group | Yes/No |
| Any other health care related service you have not reported in other questions. | Yes/No |
| *For each service marked “Yes” above, the following questions are presented* |  |
| Altogether, during the LAST 4 MONTHS, how many times did [name] have a visit for [service]? | 1 time; 2 times; 3 times; 4 times; 5 times; More than 5 times |
| Altogether during the LAST 4 MONTHS, about how much did your family pay for [name]'s [service]? | *Open-ended response* |
| During the LAST 4 MONTHS, has [name] had a visit for any of the following complementary or alternative medical services? |  |
| Acupuncture | Yes/No |
| Chiropractor | Yes/No |
| Naturopath | Yes/No |
| Traditional Chinese Medicine provider | Yes/No |
| Other complementary or alternative medical care service | Yes/No |
| *For each complementary or alternative medical service marked “Yes” above, the following questions are presented* |  |
| Altogether, during the LAST 4 MONTHS, how many times did [name] have a visit for [complementary or alternative medical service]? | 1 time; 2 times; 3 times; 4 times; 5 times; More than 5 times |
| Altogether during the LAST 4 MONTHS, about how much did your family pay for [name]'s [complementary or alternative medical service]? | *Open-ended response* |
| During the LAST 4 MONTHS, has [name] taken ANY medicines prescribed by a doctor or health care provider? | Yes/No |
| Please list the prescription medicines [name] has taken during the LAST 4 MONTHS. | *Open-ended response* *text boxes provided. Up to 10 entries allowed.* |
| *The following questions are only presented to respondents in the ASD and/or asthma groups. The appropriate health condition appears in the response options in REDCap.* |  |
| For each prescription medication [name] took during the LAST 4 MONTHS, please indicate if it was related to [name]'s [Autism / Asthma].  Prescription – [Filled in by REDCap with each medication name provided above] | Yes, related to [name]'s [Autism/Asthma]; No, not related to [name]'s [Autism/Asthma] |
| Altogether, about how much did your family pay for [name]'s prescription medicines during the LAST 4 MONTHS? | *Open-ended response* |
| Some children take vitamins, supplements, or herbal medicines to help with their health or chronic health conditions. Sometimes these are suggested by a medical care provider and sometimes parents try these on their own. Before you start this section, it may be helpful to get out your child's vitamin, supplement, or herbal medicine containers, since we'll ask about what your child is taking.  During the LAST 4 MONTHS, has [name] taken ANY vitamins, supplements, or herbal medicines to help with health or chronic health conditions? | Yes/No |
| Please list the vitamins, supplements, or herbal medicines [name] has taken during the LAST 4 MONTHS. | *Open-ended response* *text boxes provided. Up to 10 entries allowed.* |
| *The following questions are only presented to respondents in the ASD and/or asthma groups. The appropriate health condition appears in the response options in REDCap.* |  |
| For each vitamin, supplement, or herbal medicine [name] took during the LAST 4 MONTHS, please indicate if it was related to [name]'s [Autism/Asthma].  Vitamin, supplement, or herbal medicine # - [Filled in by REDCap with each vitamin, supplement, or herbal medicine name provided above] | Yes, related to [name]'s [Autism/Asthma]; No, not related to [name]'s [Autism/Asthma] |
| Altogether, about how much did your family pay for [name]'s vitamins, supplements, and herbal medicines during the LAST 4 MONTHS? | *Open-ended response* |
| Sometimes children need temporary or long-term medical equipment to help with their health or chronic health condition. For example, a child with a broken leg might need crutches or a child with asthma might need a home nebulizer.  During the LAST 4 MONTHS, has [name] used ANY type of medical equipment, such as a wheelchair, home nebulizer, or a blood glucose monitor? | Yes/No |
| Please list the medical equipment [name] has used during the LAST 4 MONTHS. | *Open-ended response* *text boxes provided. Up to 10 entries allowed.* |
| *The following questions are only presented to respondents in the ASD and/or asthma groups. The appropriate health condition appears in the response options in REDCap.* |  |
| For each type of medical equipment [name] used during the LAST 4 MONTHS, please indicate if it was related to [name]'s [Autism/Asthma].  Medical equipment type # – [Filled in by REDCap with each medical equipment provided above] | Yes, related to [name]'s [Autism/Asthma]; No, not related to [name]'s [Autism/Asthma] |
| Altogether, about how much did your family pay for [name]'s medical equipment during the LAST 4 MONTHS? | *Open-ended response* |

**Section 4. At Home Care**

| **Question** | **Response options** |
| --- | --- |
| ***The next questions are about things you do at home to help with your child's health or chronic health condition.*** |  |
| During the LAST 4 MONTHS, was [name] supposed to take medication or use medical equipment prescribed by a doctor or other health care provider regularly (every day or every week)? | Yes/No |
| During the LAST 4 MONTHS, about how much time did you spend helping [name] take their medication or use medical equipment in a typical week? | Less than 1 hour; 1 hour; 2 hours; 3 hours; 4 hours; 5 hours; More than 5 hours |
| During the LAST 4 MONTHS, have you used any behavior modification or other therapies suggested by a doctor or health care professional with [name]? For example, using positive reinforcement techniques, or using materials provided by doctor or therapist. | Yes/No |
| During the LAST 4 MONTHS, about how many hours did you spend using behavior modification or other therapies in a typical week? | Less than 1 hour; 1 hour; 2 hours; 3 hours; 4 hours; 5 hours; More than 5 hours |
| During the LAST 4 MONTHS, have you spent time helping [name] with physical therapy or other exercises that were suggested by a doctor or health care professional? | Yes/No |
| During the LAST 4 MONTHS, about how many hours did you spend helping with physical therapy or exercises in a typical week? | Less than 1 hour; 1 hour; 2 hours; 3 hours; 4 hours; 5 hours; More than 5 hours |
| During the LAST 4 MONTHS, did you spend any time doing paperwork or other administrative tasks (such as talking with insurance companies or filling out legal documents) related to [name]'s health or chronic health condition? | Yes/No |
| Altogether, in the LAST 4 MONTHS, about how many hours did you spend doing administrative work? | Less than 1 hour; 1 hour; 2 hours; 3 hours; 4 hours; 5 hours; More than 5 hours |
| During the LAST 4 MONTHS, did you spend any time educating or explaining to [name] or others (such as family, teachers) about [name]'s health or chronic health conditions? For example, explaining about or discussing diagnosis, symptoms or treatments. | Yes/No |
| Altogether, in the LAST 4 MONTHS, about how many hours did you spend educating or explaining about [name]'s health or chronic health condition? | Less than 1 hour; 1 hour; 2 hours; 3 hours; 4 hours; 5 hours; More than 5 hours |
| During the LAST 4 MONTHS, did you spend any time doing general research about [name]'s health or chronic health condition? This includes things like searching the internet or going to the library or bookstore. | Yes/No |
| Altogether, in the LAST 4 MONTHS, about how many hours did you spend doing research related to [name]'s health or chronic health condition? | Less than 1 hour; 1 hour; 2 hours; 3 hours; 4 hours; 5 hours; More than 5 hours |
| During the LAST 4 MONTHS, did you spend time doing any other activities related to [name]'s health or chronic health condition? | Yes/No |
| What were those activities? | *Open-ended response* |
| Altogether, in the LAST 4 MONTHS, about how many hours did you spend doing these other activities related to [name]'s health or chronic health condition? | Less than 1 hour; 1 hour; 2 hours; 3 hours; 4 hours; 5 hours; More than 5 hours |
| ***Next, we will ask you about money you might have spent to manage your child's health or chronic health condition at home.*** |  |
| During the LAST 4 MONTHS, have you paid money to anyone to help with care at home related to [name]'s health, or chronic health condition? For example, someone to help with medical equipment. | Yes/No |
| Altogether, in the LAST 4 MONTHS, about how much money did you spend for this help? | *Open-ended response* |
| During the LAST 4 MONTHS, did you spend money on safety modifications or changes to your home related to [name]'s health or chronic health condition? For example, adding a ramp for a wheelchair. | Yes/No |
| Altogether, in the LAST 4 MONTHS, about how much money did you spend on these changes? | *Open-ended response* |
| During the LAST 4 MONTHS, have you paid money to anyone to help with paperwork or legal issues related to [name]'s health or chronic health condition? For example, someone to help your child at school or in court, or a professional to help with care planning. | Yes/No |
| Altogether, in the LAST 4 MONTHS, about how much money did you spend for this help? | *Open-ended response* |
| During the LAST 4 MONTHS, did you spend money on any other activities related to [name]'s health or chronic health condition? | Yes/No |
| What were these activities? (Please describe) | *Open-ended response* |
| Altogether, in the LAST 4 MONTHS, about how much money did you spend on these other activities? | *Open-ended response* |

**Section 5. Family finances**

| **Question** | **Response options** |
| --- | --- |
| ***Next, we will ask a few questions about health insurance and other resources your family may have used to help care for your child's health or chronic health condition.***  ***Remember, the period of time you will be answering about is the 4-month period beginning around [date calculated by REDCap].*** |  |
| During the LAST 4 MONTHS, was [name] covered by ANY type of health insurance that helped pay (or would have helped pay) for medical care such as doctor's visits? | Yes/No |
| During the LAST 4 MONTHS, what type of health insurance did [name] have? | ***Check all that apply.***  Private insurance through parent's job or employer (such as Aetna, Blue Cross, Kaiser, Providence, United Health); Private insurance purchased by family (such as Aetna, Blue Cross, Kaiser, Providence, United Health, insurance purchased through Affordable Care Act); Public insurance (such as Medicaid, Med Quest, Oregon Health Plan, Medi-Cal, Apple Health or other public insurance); Insurance through parent's Military Service (such as TRICARE, CHAMPUS); Indian Health Service; Other insurance; None of the above |
| During the LAST 4 MONTHS, how often did [name]'s health insurance offer benefits or cover services that met their needs? | Always; Usually; Sometimes; Never |
| During the LAST 4 MONTHS, how often did [name]'s health insurance allow them to see the health care providers he or she needed? | Always; Usually; Sometimes; Never |
| During the LAST 4 MONTHS, what was the combined monthly income for all members of your family before taxes, in a typical month?  Please include all sources of income such as money from jobs, social security, child or spousal support, unemployment, and public assistance, or other income. If you have had the same income all year, you can divide your family's yearly income by 12 to get the amount for a typical month. | Less than $1,000 per month; $1,001-2,000 per month; $2,001-4,000 per month; $4,001-6,000 per month; $6,001-8,000 per month; $8,001-12,000 per month; $12,001-15,000 per month; $15,001-20,000 per month; More than $20,000 per month |
| During the PAST 4 MONTHS, did your family have problems paying for any medical care, caregiving, or other services related to your child's health or chronic health condition? | Yes/No |
| During the LAST 4 MONTHS, did your family use any of the following to help pay for [name]'s medical care, caregiving, or other services related to [name]'s health or chronic health condition? | ***Check all that apply.***  We used savings; We used credit cards; We paid for care with a payment plan that allows us to pay over time; We cut down on spending for basics (for example food and/or clothing); We cut down on recreational activities (for example eating out, movies, entertainment); We sold possessions or property (for example car, jewelry, TV, computer); We borrowed money from a bank, family, friends, or another type of loan; We changed our living situation (for example moved to a less expensive house or apartment); Other; None of the above |
| During the PAST 4 MONTHS, did you choose not to seek medical care or other services for [name] because your family couldn't afford to pay for it? | Yes/No |
| During the PAST 4 MONTHS, how would you rate your level of distress due to the costs of medical care, caregiving or other services related to [name]'s health or chronic health condition? | Not at all distressed; Slightly distressed; Moderately distressed; Very distressed; Extremely distressed |

**Section 6. Employment**

| **Question** | **Response options** |
| --- | --- |
| ***Next, we will ask questions about whether or not [name]'s health or chronic health condition has affected your work.*** |  |
| During THE LAST 4 MONTHS, did you work at all at a job for money or as part of your own business? | Yes/No  *[The response of “Yes” or “No” determines which questions below the respondent receives]* |
| You indicated that you did not work at all for money during the LAST 4 MONTHS. What best describes your situation? | ***Check all that apply.***  Taking care of my home or family; Looking for work; Could not find work; Could not work because of child's health or chronic health condition; Unable to work because I am disabled; On temporary layoff from a job; Maternity/Paternity leave; Going to school; Wanted some time off; Retired; Other; None of the above |
| During the LAST 4 MONTHS, did you work for money at more than one job? | Yes/No |
| Which category best describes the organizations or businesses you worked for during the LAST 4 MONTHS? | ***Check all that apply.***  Self-employed or own business; Work for a private company or business (for example, a hospital, grocery store); Work for a public organization (for example, the police, post office, a public school); None of the above |
| Which category or categories best describe the jobs you had during THE LAST 4 MONTHS? | ***Check all that apply.***  Management/professional and technical (for example, manager, engineer, architect, accountant, teacher, nurse, doctor, lawyer); Technical support (computer support, health technician, science technician); Sales or Marketing (retail clerk, sales representative, insurance agent, real estate broker); Administrative (clerk, secretary, data processor, telephone operator); Service (security guard, housekeeping, food service, nurse aide, janitor); Production, construction, or operations (mechanic, carpenter, plumber, machine operator); Transportation (motor vehicle operator, truck driver, bus driver, moving equipment operator); Other job; None of the above |
| Altogether, during the LAST 4 MONTHS, how many weeks did you work at all? | The entire 4 months; 1 week; 2 weeks; 3 weeks; 4 weeks; 5 weeks; 6 weeks; 7 weeks; 8 weeks; 9 weeks; 10 weeks; 11 weeks; 12 weeks; 13 weeks; 14 weeks; 15 weeks |
| During the LAST 4 MONTHS, about how many hours did you work in a typical work week? | Less than 5 hours; 5 hours; 10 hours; 15 hours; 20 hours; 25 hours; 30 hours; 35 hours; 40 hours; 45 hours; 50 hours; 55 hours; 60 hours; A different number of hours |
| What categories best describe how you were paid for work during the LAST 4 MONTHS? | ***Check all that apply.***  Paid by the hour (hourly wage); Salary; Commission; Piece work; Other; None of the above |
| During the LAST 4 MONTHS, did the management practices in your workplace make it easy or difficult for you to deal with problems related to your child's health or chronic health condition during working hours? | Very easy; Easy; Somewhat easy; Somewhat difficult; Difficult; Very difficult |
| ***Next, we will ask how your child's health or chronic health condition might have affected your work during the workday.*** |  |
| During the LAST 4 MONTHS, have you experienced any of the following job changes because of [name]'s health or chronic health condition? | ***Check all that apply.***  Quit job; Fired from job; Changed job; Changed work schedule; Reduced number of hours worked; Increased number of hours worked; Retired early; Other; None of the above |
| During the LAST 4 MONTHS, have you experienced any of the following situations at work because of [name]'s health or chronic health condition? | ***Check all that apply.***  Taken whole days off; Been late for work; Left work during the workday or left work early; Been interrupted by phone calls or other matters related to your child's health or chronic health condition during work hours; Other; None of the above |
| During the LAST 4 MONTHS, do you think [name]'s health or chronic health condition ever affected how you spent your time or how well you focused or performed tasks while at work? | Yes/No |
| During the LAST 4 MONTHS, about how often do you think [name]'s health or chronic health condition affected how you spent your time or how well you focused or performed tasks while at work? | Almost all the time; Often; Sometimes; Once in a while; Rarely |

**References**

1. Lynch FL, Vuckovic N, Schneider J, Firemark AJ: **Development of and preliminary results of a survey instrument to measure the family costs associated with child mental health conditions**. In: *6th International Health Economics Association (IHEA) World Congress: July 8-11 2007; Copenhagen, Denmark*; 2007.

2. Lynch F, Dickerson J: **Validity and Reliability of the Family Economic Impact Interview: A New Instrument to Measure Costs Related to Child Mental Health Conditions (abstract)**. *Clinical Medicine & Research* 2012, **10**(3):181.
