# Supplementary material for: A framework for measuring the cost to families of caring for children’s health: the design, methodology, and study population of the r-Kids study
Source: BMC Pediatr. 2023 Mar 20;23:128. doi: 10.1186/s12887-023-03893-7 (PMC10025806; doi:10.1186/s12887-023-03893-7)
Supplement: Supplementary file 2 — Additional file 2: SupplementalTable 1. Multivariable Regression Analysis Parameters Estimates (PE) andStandard Errors (SE) of Child and Survey Respondent Mental Health, PhysicalHealth, and Quality of Life. [file 12887_2023_3893_MOESM2_ESM.docx]

**Supplemental Table 1. Multivariable Regression Analysis Parameters Estimates (PE) and Standard Errors (SE) of Child and Survey Respondent Mental Health, Physical Health, and Quality of Life**

|  | **Psychosocial health summary score (PedsQL)** | | **Physical health summary score (PedsQL)** | | **Externalizing score (SDQ)** | | **Internalizing score (SDQ)** | | **Prosocial score (SDQ)** | | **Parenting stress score** | | **Respondent overall physical health** | | **Respondent overall mental health** | |
| --- | --- | --- | --- | --- | --- | --- | --- | --- | --- | --- | --- | --- | --- | --- | --- | --- |
|  | ***PE (SE)*** | ***p*** | ***PE (SE)*** | ***p*** | ***PE (SE)*** | ***p*** | ***PE (SE)*** | ***p*** | ***PE (SE)*** | ***p*** | ***PE (SE)*** | ***p*** | ***PE (SE)*** | ***p*** | ***PE (SE)*** | ***p*** |
| Intercept | 76.57 (0.94) | <.001 | 87.23 (1.17) | <.001 | 3.89 (0.23) | <.001 | 5.61 (0.25) | <.001 | 8.29 (0.14) | <.001 | 34.98 (0.65) | <.001 | 3.47 (0.06) | <.001 | 3.67 (0.06) | <.001 |
| Study Group |  |  |  |  |  |  |  |  |  |  |  |  |  |  |  |  |
| *ASD* | -22.7 (1.0) | <.001 | -15.86 (1.24) | <.001 | 4.82 (0.24) | <.001 | 4.62 (0.26) | <.001 | -2.89 (0.14) | <.001 | 6.18 (0.69) | <.001 | -0.16 (0.07) | .015 | -0.24 (0.07) | .001 |
| *Asthma* | -1.4 (1.04) | .177 | -5.2 (1.29) | <.001 | 0.13 (0.25) | .602 | 0.57 (0.27) | .033 | 0.06 (0.15) | .664 | -0.36 (0.71) | .613 | -0.16 (0.07) | .014 | -0.11 (0.07) | .111 |
| *Control* | Ref. | -- | Ref. | -- | Ref. | -- | Ref. | -- | Ref. | -- | Ref. | -- | Ref. | -- | Ref. | -- |
| Age |  |  |  |  |  |  |  |  |  |  |  |  |  |  |  |  |
| *3-5* | 6.74 (1.05) | <.001 | 5.95 (1.31) | <.001 | -1.4 (0.25) | <.001 | 0.3 (0.28) | .278 | -0.7 (0.15) | <.001 | 0.37 (0.73) | .610 | 0.03 (0.07) | .653 | 0.01 (0.07) | .868 |
| *6-11* | 0.08 (0.94) | .928 | -0.39 (1.17) | .739 | 0.53 (0.22) | .018 | -1.01 (0.24) | <.001 | -0.07 (0.14) | .622 | -0.48 (0.65) | .454 | 0.0 (0.06) | .988 | -0.02 (0.06) | .757 |
| *12-17* | Ref. | -- | Ref. | -- | Ref. | -- | Ref. | -- | Ref. | -- | Ref. | -- | Ref. | -- | Ref. | -- |
| Gender |  |  |  |  |  |  |  |  |  |  |  |  |  |  |  |  |
| *Female* | -0.45 (1.0) | .652 | -2.14 (1.24) | .085 | 0.18 (0.24) | .459 | -0.74 (0.26) | .005 | 0.42 (0.15) | .004 | 0.43 (0.69) | .533 | -0.06 (0.06) | .339 | 0.05 (0.07) | .453 |
| *Male* | Ref. | -- | Ref. | -- | Ref. | -- | Ref. | -- | Ref. | -- | Ref. | -- | Ref. | -- | Ref. | -- |
